# Medical Botany; or, Descriptions of the More Important Plants Used in Medicine, with Their History, Properties, and Mode of Administration

**Published:** 1847-07

**Authors:** 


Art. VI.—*Medical Botany: or Descriptions of the more important Plants used in Medicine, with their History, Properties, and Mode of Administration.* By R. Egglesfeld Griffith, M D., Member of the American Philosophical Society, of the Academy of Natural Sciences of Philadelphia, etc. etc. With upwards of three hundred Illustrations. *"Scire potestates herbarum usumque medendi."* Æneid xii, 396. 8vo, pp, 704. Philadelphia: Lea and Blanchard: 1847.

" It may be asked," says the author of this treatise, in his preface, " will not the excellent works of Pereira, Royle, Ballard, and Garrod, and Drs. Wood and Bache, supply all the botanic knowledge that is required by the student?" He thinks not, and with that impression has prepared his work "as a companion to the more practical treatises," embracing in it matter relative to the systematic classification, characters and history of medical plants which those works do not contain.

The title of this work is attractive, and its appearance equally so; its numerous illustrations—more than three hundred—give to it an air of beauty and taste well calculated to prepossess one in its favor. We hope it may allure the medical youth of our country to the much neglected study of botany.

It begins with an introductory chapter on the *anatomy* of plants, our knowledge of which has been wonderfully extended by the microscope. The student will consult this portion of the work with profound interest, and will find here, if he has never turned his attention to the subject before, a new world full of wonders.

Vegetable chemistry is also touched upon in this chapter, and the various medicinal products of plants are briefly referred to.

To this introduction succeeds a definition of the technical terms used in botany, or *botanical terminology*—a considerate labor, for which the author is entitled to the thanks of every student of his work. He includes medical plants under six classes and fifty-six groups, and it is very evident that one who expects to profit by his descriptions of them must sit down to the diligent study of his treatise. Both for the pupil about to enter upon the study, and the practitioner who may wish to refresh his memory on any point of medical botany, Dr. Griffith will be found to have performed a useful and acceptable office in the preparation of this manual.

This work, as well as the others herein noticed, may be had of Messrs. Maxwell and Prescott, booksellers in this city, whose promptness in placing upon our table all the new publications in medicine has brought us under many obligations.

